# Selective inhibition of phosphodiesterases 4, 5 and 9 induces HSP20 phosphorylation and attenuates amyloid beta 1–42‐mediated cytotoxicity

**DOI:** 10.1002/2211-5463.12156

**Published:** 2016-12-05

**Authors:** Ryan T. Cameron, Ellanor Whiteley, Jon P. Day, Anna I. Parachikova, George S. Baillie

**Affiliations:** ^1^ Institute of Cardiovascular and Medical SciencesCollege of Veterinary Medical and Life SciencesUniversity of GlasgowUK; ^2^ Synaptic TransmissionH. Lundbeck A/SValbyDenmark

**Keywords:** Alzheimer's disease, beta amyloid, cyclic AMP, cyclic GMP, heatshock protein 20, phosphodiesterase

## Abstract

Phosphodiesterase (PDE) inhibitors are currently under evaluation as agents that may facilitate the improvement of cognitive impairment associated with Alzheimer's disease. Our aim was to determine whether inhibitors of PDEs 4, 5 and 9 could alleviate the cytotoxic effects of amyloid beta 1–42 (Aβ_1–42_) via a mechanism involving the small heatshock protein HSP20. We show that inhibition of PDEs 4, 5 and 9 but not 3 induces the phosphorylation of HSP20 which, in turn, increases the colocalisation between the chaperone and Aβ_1–42_ to significantly decrease the toxic effect of the peptide. We conclude that inhibition of PDE9 is most effective to combat Aβ_1–42_ cytotoxicity in our cell model.

**Abbreviations**
AD: Alzheimer's disease
Aβ: amyloid beta
DMEM: Dulbecco's Modified Eagle's Medium
HSP20: heatshock protein 20
PDE: phosphodiesterase
PKA: protein kinase A
PKG: protein kinase G

Alzheimer's disease (AD) is the most common form of dementia in the elderly and numerous studies have shown the amyloid beta (Aβ) peptide to be a key toxic component in AD. Involvement of soluble or higher order aggregation species of the Aβ peptide, in various intra‐ or extracellular compartments, has been linked to neurotoxicity. However, the underlying toxic mechanism triggered by Aβ dyshomeostasis promoting the loss of neurons and synaptic failure is still being investigated 1.

One signalling pathway that has been targeted to improve synaptic function is the cyclic nucleotide second messenger system. Cyclic AMP (cAMP) 2 and cyclic GMP (cGMP) 3 signalling systems have been shown to have a crucial input into regulation of synaptic plasticity, learning and memory. As a result, signalling intermediates within these pathways such as phosphodierases 4 and cAMP response element‐binding protein (CREB) 5 have been identified as possible therapeutic targets for AD.

The superfamily of phosphodiesterases (PDEs) is an attractive target for modulating synaptic plasticity via second messenger signalling as these enzymes provide the sole means of cyclic nucleotide degradation. There are 21 different genes that encode PDEs and these are separated functionally into 11 families depending on characteristics such as cyclic nucleotide specificity and modular structure 6. Further diversity is generated through multiple splice variants existing for a number of PDE families resulting in more than 60 different isoforms of PDE 6. A number of PDEs have been associated with signalling pathways involved in neuropsychiatric disorders, particularly PDE4, PDE5 and PDE9, with the latter two more recently emerging as novel therapeutic targets for AD 7.

A topical neuroprotective mechanism that is a consequence of the inhibition of PDE4 and PDE5 is the hyperphosphorylation of the molecular chaperone heatshock protein 20 (HSP20) on serine 16 by protein kinase A (PKA) or protein kinase G (PKG) 8. HSP20 is known to associate with the pathological hallmarks of AD brains 9 and to physically bind to the Aβ peptide, reducing its toxicity to neurons 10. Recent data from our lab have shown that inhibition of a PDE4 pool directly associated with HSP20 results in its increased phosphorylation by PKA 11 which in turn promotes association of the chaperone with the self association domain of the Aβ peptide, preventing its oligomerisation 12 and reducing its cytotoxicity 13. We now investigate whether inhibition of PDEs 5 and 9 result in similar functional consequences with respect to HSP20 phosphorylation and Aβ toxicity in a cellular model. We report that both inhibition of PDE5 and PDE9 result in a dose‐dependent phosphorylation of HSP20 on serine 16 that promotes greater association of HSP20 with Aβ to decrease Aβ‐induced cell death. Our results suggest a novel mechanism for PDE inhibitors in the context of AD.

## Materials and methods

### Materials

All chemicals used to conduct this research were of analytical grade and were supplied by Sigma‐Aldrich, Poole, UK, unless otherwise stated. Compounds were typically dissolved in DMSO and added to cells at a concentration no greater than 0.1% (v/v). All aqueous solutions were prepared with deionised water (dH_2_O; Millipore, Livingston, UK). Antibodies used in the phosphorylation assays and ICC, α‐pS16‐HSP20 Abcam (ab58522; Cambridge, UK), α‐Rabbit‐HRP Sigma‐Aldrich (A6154), α‐Rabbit‐Alexa Fluor^®^ 594 Invitrogen (Paisley, UK; A‐11012). Primary antibody used for PDE9 western blotting experiment was supplied by Scottish Biomedical (Glasgow, UK).

### RT‐PCR

Approximately 10^6^ SHS5Y cells were harvested, washed in PBS, then snap‐frozen and stored at −80 °C. RNA was prepared using RNeasy column (Qiagen, London, UK) with on‐column DNAse digestion (Qiagen). About 1.5 μg of total RNA (1 μL) was used to synthesise 20 μL of cDNA using Superscript Vilo cDNA synthesis kit (Invitrogen) with incubation times as follows: 25 °C, 10 min; 42 °C, 120 min; 85 °C, 15 min. PDE9A1 intron‐spanning RT‐PCR primer pairs were designed to three separate regions of the gene sequence (PDE9A1_1, PDE9A1_2, PDE9A1_3), and were resuspended to a stock concentration of 10 μmol·L^−1^ in nuclease‐free water. RT‐PCR reactions were performed using PDE9A1 primers and GAPDH primers as a positive control at a concentration of 200 nmol·L^−1^. For the PCR, pre‐mixed PCR reagent (Thermo Scientific, Paisley, UK) was used with the following thermal cycling parameters: 94 °C, 2 min then 30 cycles of 94 °C, 30 s; 55 °C, 30 s; 72 °C, 30 s, followed by 72 °C for 20 min. One microlitre of cDNA was used as a template in each 20‐μL reaction. No template control reactions lacking cDNA were included to ensure primer contamination had not occurred. Ten microlitres of each PCR product was resolved on a 1.8%, 0.5× TBE agarose gel. Bands corresponding to the predicted molecular weight were obtained for each PCR reaction strongly suggesting that PDE9A1 mRNA is expressed in SHS5Y cells.

### Preparation of Aβ

For cell‐based assays, synthetic Aβ peptides were purchased from rPeptide^®^ (Athens, GA, USA). Aβ1–42 (A‐1002) peptides are the recombinant form of the human Aβ peptide. Aβ1–42 scrambled peptide (Aβscr; A‐1004) which is a rearranged version of the peptide that carries the overall weight and charge of Aβ1–42, was used as a control. For ICC experiments, fluorescein‐tagged Aβ1–42 was purchased from Anaspec (ANA23525‐05; Fremont, CA, USA). Peptides were dissolved in DMSO at a concentration of 5 mg·mL^−1^ and sonicated in a water bath for 15 min. Samples were aliquoted and stored at −20 °C until required. To create neurotoxic Aβ1–42 derivatives a method similar to that described by Lambert *et al*. 14 was used, where Aβ1–42 (or scrambled) peptides were brought to 100 μm in cold PBS and incubated at 4–8 °C for 24 h. The resulting aggregated peptides were added directly to cell culture medium typically at 1 : 10 dilution (Aβ : media). Samples from each 100‐μm stock added were taken for SDS/PAGE and western blotting analysis.

### Western blotting

The SDS/PAGE gels were transferred to nitrocellulose membranes using an Invitrogen X‐Cell apparatus (Invitrogen) using Nupage^®^ X‐cell Blotting Module and 20× NuPage^®^ transfer buffer containing 20% methanol (v/v) in 200 mL of dH_2_O. Proteins were transferred at 28 V for 1.5 h and transfer efficacy was established through visualisation of molecular weight markers or Ponceau staining. Following transfer membranes were incubated in 5% milk solution (w/v) in 1× TBST (20 mm Tris‐Cl pH7.6, 150 mm NaCl, 0.1% Tween 20) for 1 h at room temperature under agitating conditions to block nonspecific antibodies binding to the membrane. Membranes were then incubated in 1% milk solution with the appropriate primary antibody added and incubated overnight at 4 °C with agitation. The membrane was then washed for 3 × 10 min with 1× TBST, and incubated in fresh 1% milk solution containing appropriate horseradish peroxidase (HRP)‐conjugated secondary antibody for 1–2 h at room temperature. After incubation, membranes were washed for 3 × 10 min before adding Pierce enhanced chemiluminescence (ECL) western blotting substrate (Thermo Scientific). Membranes were incubated in ECL substrate for 1 min before transferring to a light‐sensitive cassette. Autoradiographic film was used to detect any signals from membranes and films were developed on a Kodak X‐omat Model 2000 processer (Rochester, NY, USA). Resulting images were quantified using quantity one (BioRad, Hemel Hempstead, UK).

### Mammalian cell culture

Cell cultures were examined using a phase contrast inverted microscope (Leitz Diavert, Midland, ON, Canada) in order to analyse the condition of the cells and to monitor for contamination. SH‐SY5Y cells were grown in Dulbecco's Modified Eagle's Medium (DMEM) and F12‐Ham's at a 1 : 1 ratio, media was supplemented with 10% (v/v) FBS, 1% (v/v) l‐Glutamine, 1% (v/v) Minimum Essential Medium with Nonessential Amino Acids (MEM‐NAA) and 1% (v/v) Penicillin–Streptomycin. Cells were cultured in a humidified, 5% (v/v) CO_2_, 37 °C incubator.

### Real‐time cell monitoring (xCELLigence)

The xCELLigence system Real‐Time Cell Analyzer RTCA‐SP (ACEA Biosciences, San Diego, CA, USA) is an electrical impedance‐based real‐time cell‐monitoring system for detection of cellular viability. The recording of cell index values (CI), normalisations and the monitoring of Aβ1–42‐mediated cytotoxicity was performed using rtca Software 1.2 (San Diego, CA, USA). The RTCA‐SP device was calibrated using RTCA Resistor Plate 96 prior to each experiment and impedance measurements were carried out in 96‐well E‐plates (ACEA). The impedance readout is expressed as arbitrary cell index values. The normalisation of cell index arbitrarily sets cell index to 1 at a desired time point, which is typically the time of adding compounds. The background impedance caused by the media is measured using 100 μL in each well prior to seeding of cells and is automatically subtracted by the rtca software using the following equation: CI − (*Ζ*_i_ − *Ζ*_o_)/15 with *Ζ*_i_ as the impedance at any given time point and *Ζ*_o_ being the background signal 15.

### HSP20 phosphorylation assays

The SH‐SY5Y cells were seeded at a density of 1 × 106 cell per well onto six‐well plates for at least 16 h prior to treatment with various PDE inhibitors. Compounds were diluted in media and added to cells for 0.5, 1, 2, 4 & 6 h for time‐course assays or incubated for 15 min for dose–response assays prior to harvesting using 3T3 lysis buffer supplemented with protease inhibitor Mini‐Complete and phosphatase inhibitor phosSTOP (Roche, Welwyn Garden City, UK). Phospho‐HSP20 levels were analysed using standard SDS/PAGE and western blotting techniques described previously.

### Immunocytochemical staining of SH‐SY5Y cells

The SH‐SY5Y cells (2 × 10^5^/well) were grown overnight in six‐well plates containing sterile coverslips. Coverslips were sterilised using an ethanol : ether solution (1 : 1, v/v) and air‐dried for a minimum 30 min in a cell culture hood. The following day cells were treated with 1 μm of fluorescein‐tagged Aβ_1–42_ (FAM‐Aβ_1–42_) and incubated for 6 h in order to mimic the real‐time cell‐monitoring assays. SH‐SY5Y cells were then treated with either rolipram (10 μm), sildenafil (1 μm), PF‐04447943 (1 μm), BAY 73‐6691 (1 μm) or DMSO only control for 1 h prior to fixation and immunostaining for phospho‐HSP20. Cells were fixed on glass coverslips using −20 °C methanol solution for 5 min, then washed twice for 5 min in cold PBS with gentle agitation. Cells were permeabilised for 20 min at room temperature with PBST (0.1% Triton‐X100 in PBS), then washed for 5 min in PBS alone. Nonspecific antibody‐binding sites were blocked by incubating with blocking buffer [0.5% BSA (w/v) in PBS] for either 30 min at room temperature, or overnight at 4 °C, coverslips were then washed twice in PBS for 5 min. The phosphor‐HSP20 primary antibody was diluted to the desired concentrations in blocking buffer, and coverslips were incubated with primary antibodies overnight at 4 °C. Coverslips were then washed three times for 10 min in PBS, and incubated with 1 : 500 dilutions of appropriate fluorescently labelled secondary antibodies in a final volume of 500 μL per coverslip and protected from light. This step took place overnight at 4 °C. Following secondary antibody incubation, coverslips were washed once in PBS and mounted onto glass slides using ProLong Gold antifade reagent with 4′,6‐diamidino‐2‐phenylindole nuclear stain (Molecular Probes, Eugene, OR, USA) and air‐dried for a minimum 48 h prior to use. Coverslips were stored at 4 °C, protected from light.

## Results

**Figure 1:**
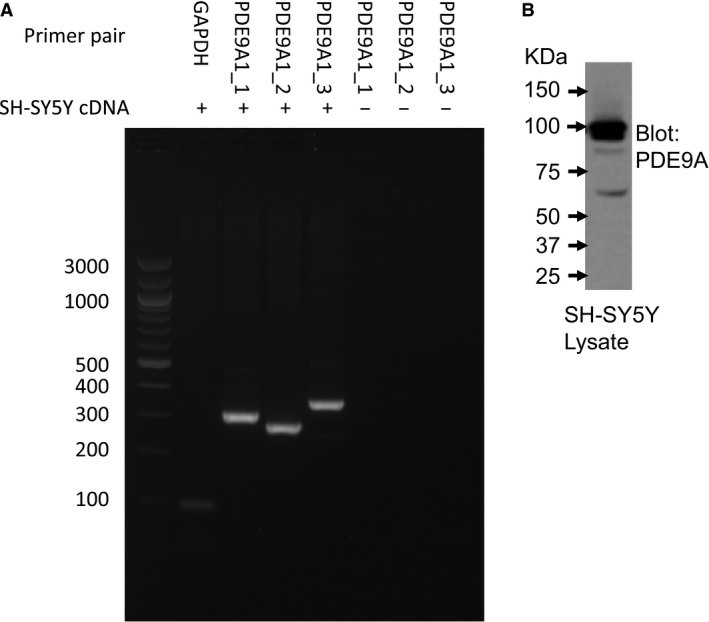
PDE9 expression in SH‐SY5Y cells. (A) RT‐PCR of SH‐SY5Y RNA using PDE9A1 intron‐spanning RT‐PCR primer pairs designed to three separate regions of the gene sequence (PDE9A1_1, PDE9A1_2, PDE9A1_3). Negative controls where no cDNA template was included are maked (−). (B) Western blot of SH‐SY5Y lysates identifying protein expression of PDE9 isoforms.

Expression of PDE4 13 and PDE5 16 had already been confirmed in our cellular model SH‐SY5Y, although there has been no such confirmation of PDE9. As a result, our first priority was to confirm the expression of PDE9 in this cell line. Firstly, we used RT‐PCR of SH‐SY5Y RNA using primer pairs targeted to three distinct intron‐spanning regions of the PDE9A1 gene (Fig. 1A). All three PDE9A primers amplified PDE9A cDNA from SH‐SY5Y cells. Secondly, we used a commercially available PDE9A antibody to positively identify the enzyme in SH‐SY5Y cell lysates. We observed PDE9A protein at a number of different molecular weights, suggesting that a number of isoforms are expressed (Fig. 1B).

**Figure 2:**
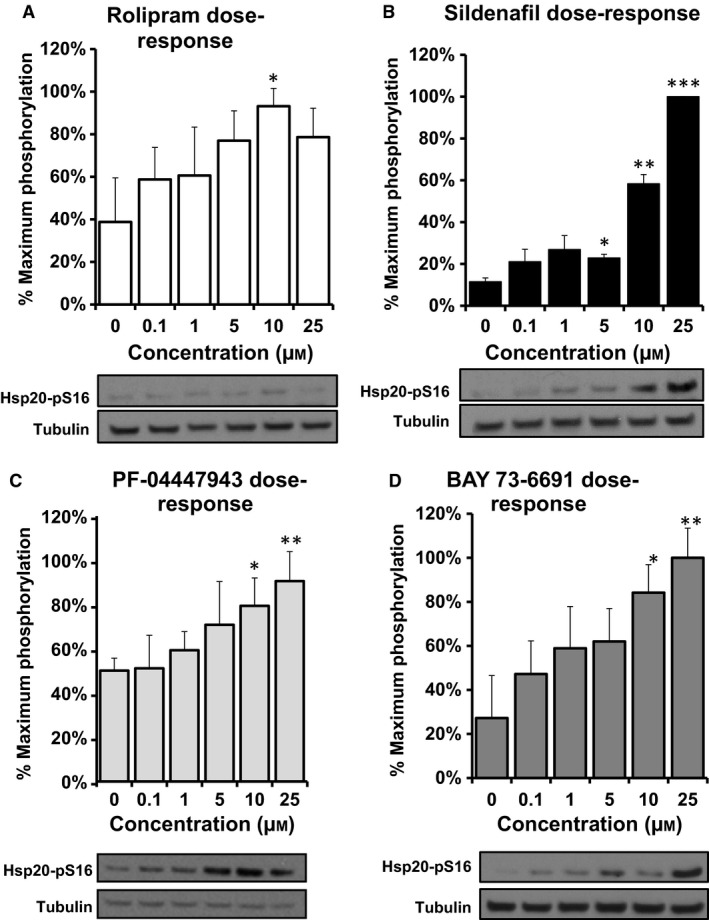
PDE inhibitors increase phosphoHSP20 levels in a time‐dependent fashion. SH‐SY5Y cells were incubated with indicated PDE inhibitors (A) Rolipram (10 μm), (B) Sildenafil (1 μm), (C) PF‐04447943 (25 μm) and (D) BAY 73‐6691 (25 μm) over a time course of 6 h. Cell lysates were prepared and blotted for phospho‐HSP20. Levels of phospho‐HSP20 were normalised against tubulin and increases plotted against control, untreated samples *n* = 3, errors are SEM. Significances were determined using Student's *t*‐test. *P = 0.05, **P < 0.01, ***P = 0.001.

**Figure 3:**
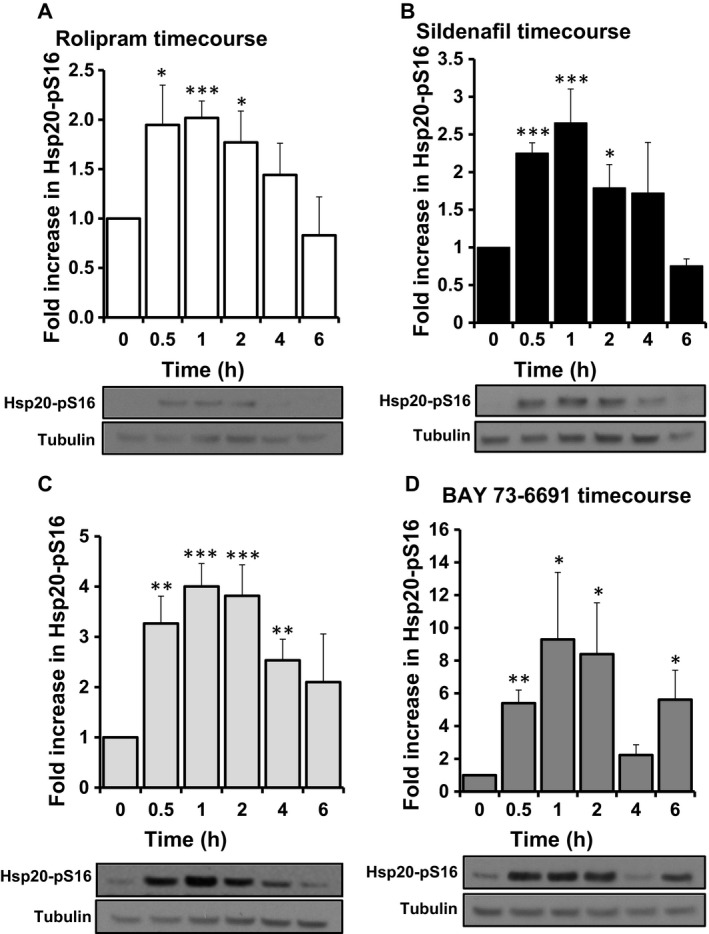
PDE inhibitors increase phospho‐HSP20 levels dose dependently. SH‐SY5Y cells were incubated with increasing concentrations of PDE inhibitors (A) Rolipram, (B) Sildenafil, (C) PF‐04447943 and (D) BAY 73‐6691 for 15 min. Cell lysates were prepared and blotted for phospho‐HSP20. Levels of phospho‐HSP20 were normalised against tubulin and increases plotted against control, untreated samples *n* = 3, errors are SEM. Significances were determined using Student's *t*‐test. *P = 0.05, **P < 0.01, ***P = 0.001.

HSP20 phosphorylation at serine 16 is induced by the inhibition of PDE4 and PDE5 via PKA and PKG activation, respectively, however, it has not been shown that PDE9 inhibition can influence such a modification of the chaperone. In light of this, we decided to compare HSP20 phosphorylation in SH‐SY5Y cells using western blotting following pharmacological inhibition of PDE4 using rolipram (Figs 2A and 3A), PDE5 with sildenafil (Figs 2B and 3B), PDE3 using cilostimide (data not shown) and PDE9 utilising two commercially available PDE9 inhibitors, PF‐04447943 (Figs 2C and 3C) and BAY 73‐6691 (Figs 2D and 3D). We lSH‐SY5Y cells were treated with various concentrations of inhibitors and incubated for 15 min (Fig. 2). All of the inhibitors (except cilostimide: data not shown) promoted dose‐dependent increases in HSP20 phosphorylation compared with the vehicle control, with 25 μm‐ treated cells being statistically significant for all inhibitors except for rolipram (Fig. 2B–D). Rolipram response was significant over control values when tested at 10 μm (Fig. 2A). In addition to dose responsiveness, we also evaluated the temporal increase in HSP20 phosphorylation over a 6‐h time‐course of each inhibitor. As previously reported, PDE4 (Fig. 3A) and PDE5 inhibition (Fig. 3B) produced a transient and significant increase in HSP20 phosphorylation, peaking at 1 h. Interestingly, this effect was also observed following treatment with both PDE9 inhibitors. PF‐04447943 treatment caused a significant fourfold increase (±0.5, *P* = 0.0001) after 1 h (Fig. 3C) compared to a ninefold increase (±4, *P* = 0.03) following BAY 73‐6691 addition (Fig. 3D) at the same time point.

**Figure 4:**
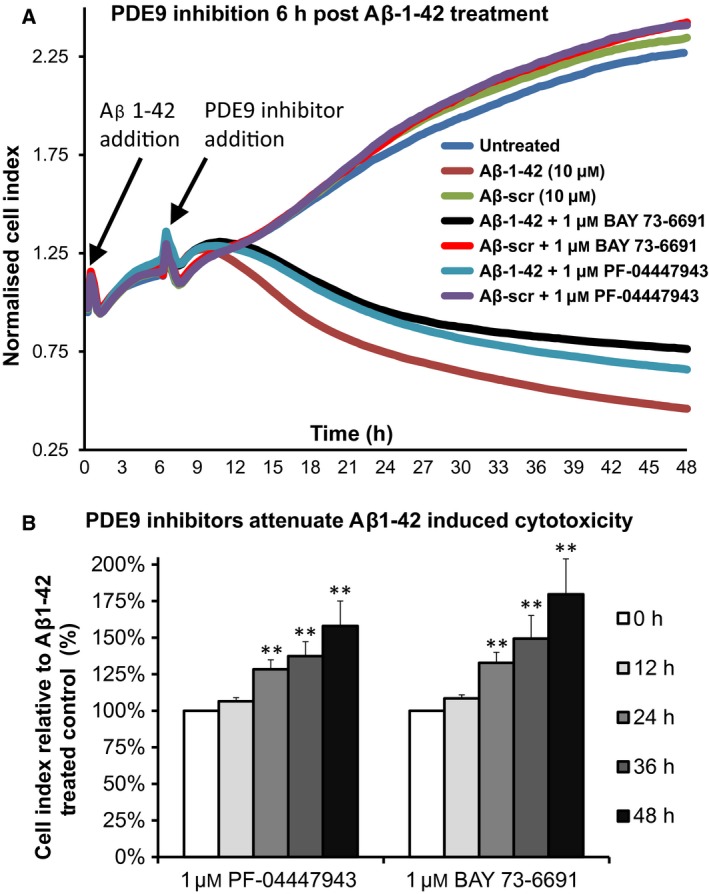
Inhibition of PDE9 significantly attenuates Aβ_1–42_‐induced cell death. (A) Noninvasive evaluation of SH‐SY5Y cell viability was undertaken using xCELLigence Real‐Time Cell Analyzer RTCA‐SP following addition of Aβ_1–42_ or a scrambled version of Aβ_1–42_ PDE 9 inhibitors were added 6 h after Aβ_1–42_ addition. (B) Evaluation of changes in normalised Cell Index (*n* = 3) over the Aβ_1–42_ time‐course following inhibition of PDE9 with PF‐04447943 (left) or BAY 73‐6691 (right). Errors are SEM and significances vs untreated controls determined using Student's *t*‐test. **P < 0.01.

As we have previously published that increases in HSP20 phosphorylation at serine 16 triggers a reduction in Aβ peptide toxicity in SH‐SY5Y cells 13, we sought to discover whether the induction of phospho‐HSP20 via pharmacological PDE inhibition (Figs 2 and 3) could recreate this neuroprotective effect. Using the xCELLigence system for label‐free, real‐time monitoring of Aβ‐induced cytotoxicity 13, preliminary growth curves demonstrated that divergence between the Aβ_1–42_ and Aβscr growth curves occurred after approximately 6 h of peptide incubation. We concluded that it takes 6 h for significant quantities of soluble Aβ_1–42_ oligomers to accumulate within the cells to induce SH‐SY5Y death, hence we added PDE inhibitors 6 h post Aβ_1–42_ addition. Treatment with either PF‐04447943 or BAY 73‐6691 induced a pronounced effect on neuroblastoma growth curves (Fig. 4A), significantly reducing the amount of cell death by Aβ_1–42._ Inhibition of PDE9 provided significant protection against the cytotoxic effects of Aβ_1–42_ as early as 12 h post treatment (Fig. 4B), reaching a maximum at 48 h, 158% (±24%, *P* = 0.016) for PF‐04447943 and 180% (±35%, *P* = 0.02) for BAY 73‐669.

**Figure 5:**
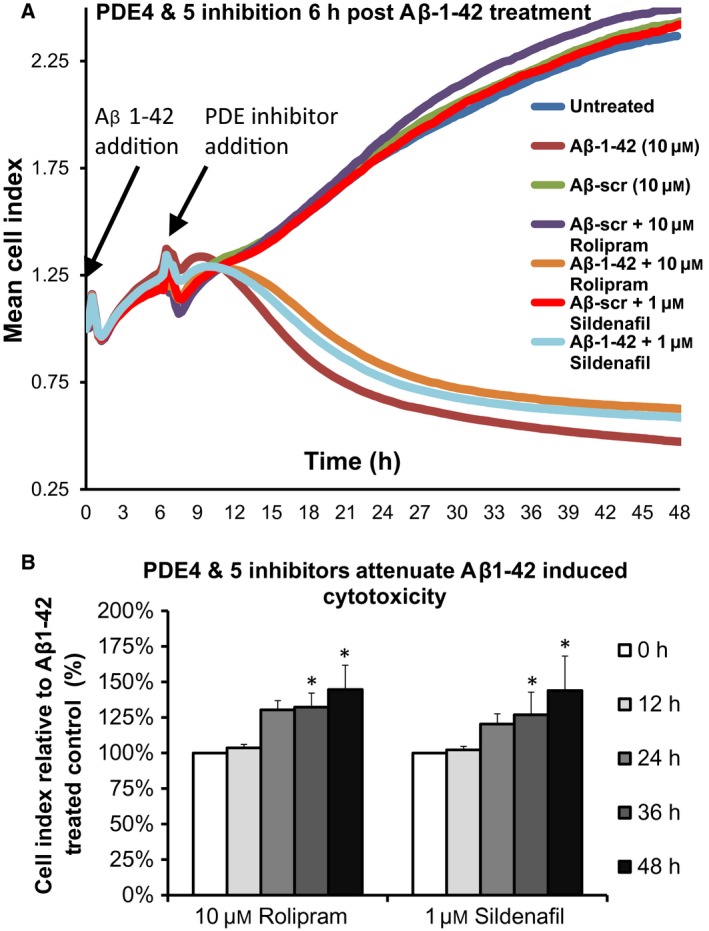
Inhibition of PDE4 and PDE5 significantly attenuates Aβ_1–42_‐induced cell death.(A) Noninvasive evaluation of SH‐SY5Y cell viability was undertaken using xCELLigence Real‐Time Cell Analyzer RTCA‐SP following addition of Aβ_1–42_ or a scrambled version of Aβ_1–42_ PDE 4 (rolipram) and 5 (sildenafil) inhibitors were added 6 h after Aβ_1–42_ addition. (B) Evaluation of changes in normalised Cell Index (*n* = 3) over the Aβ_1–42_ time‐course following inhibition of PDE9 with rolipram (left) or sildenafil (right). Errors are SEM and significances vs untreated controls determined using Student's *t*‐test. *P = 0.05.

When tested in the same manner, the influence of PDE4 and PDE5 inhibition on Aβ_1–42_ cytotoxicity was observed to be similar in trend but less pronounced than that promoted by PDE9 inhibition (Fig. 5A). The increase in Cell Index relative to Aβ_1–42_ control peaked at 48‐h time‐points for both rolipram and sildenafil (Fig. 5B) with maximum increases of 145% (±18%, *P* = 0.018) and 144% (±24%, *P* = 0.043) respectively (Fig. 5B).

**Figure 6:**
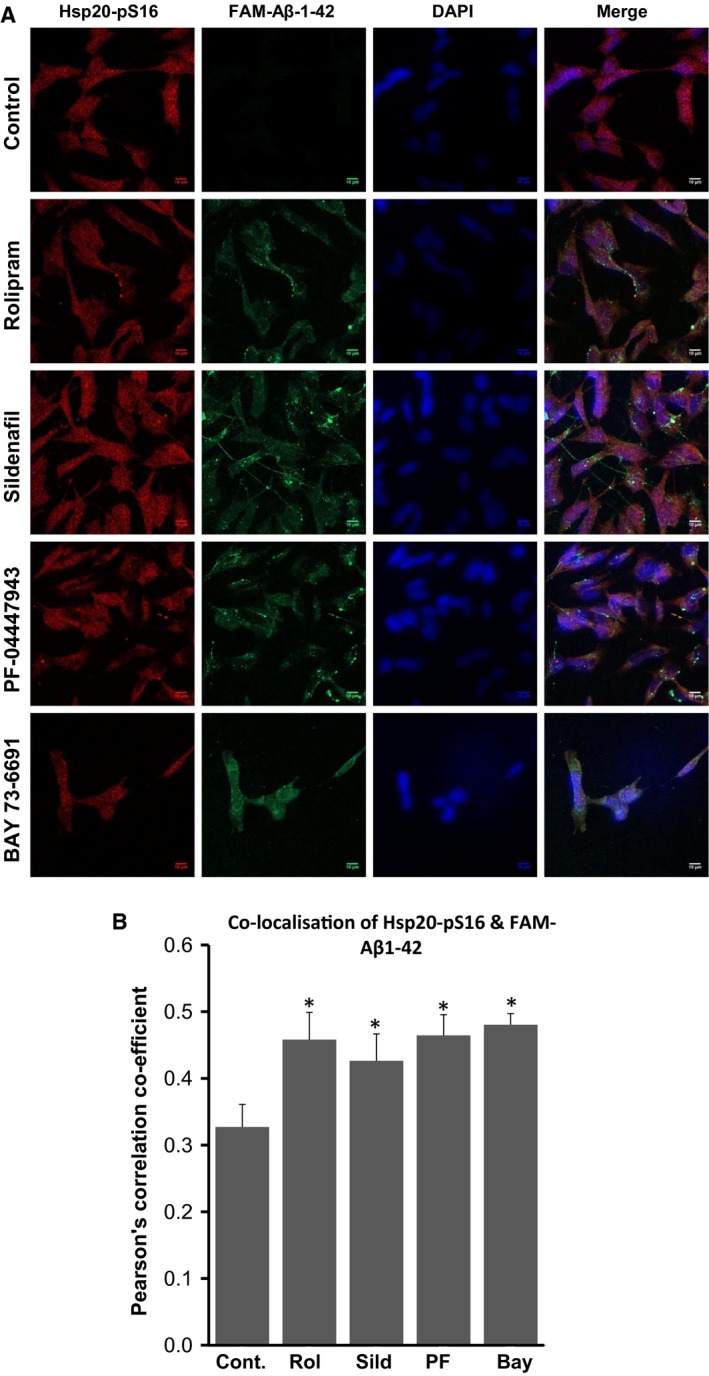
Phosphodiesterase inhibitors increase colocalisation of HSP20 and in SH‐SY5Y cells. (A) SH‐SY5Y cells were incubated with either 10 μm rolipram (Rol), 1 μm sildenafil (Sil), 1 μm PF‐04447943 (PF) or 1 μm BAY73‐6691 (BAY) for 15 min prior to methanol fixation and staining with antibodies against phospho‐HSP20 (pS16‐HSP20) and the C‐terminal domain of APP (APP‐CTD) which contains the Aβ epitope. Scale bar = 10 μm. (B) Pearson's Correlation coefficient (PCC) for the colocalisation of phosphor‐HSP20 and Aβ were calculated in cells treated with PDE inhibitors. Student's *t*‐test was used to evaluate changes compared to DMSO only controls. (*P < 0.05, Student *t*‐test, *n* = 3).

Given that selective inhibition of PDEs 4, 5 and 9 significantly protected neuronal‐like SH‐SY5Y cells against Aβ_1–42_‐induced cell death, it was important to establish if this protective mechanism was mediated to some extent through HSP20. As we have shown that phosphorylation of HSP20 increases its association with Aβ_1–42_ and reduces the effective concentration of the chaperone required to inhibit Aβ_1–42_ oligomerisation 13, we evaluated the formation of the HSP20‐fluorescein Aβ_1–42_‐tagged complex in cells using immunofluorescence (Fig. 6A). A synthetic FAM‐Aβ_1–42_ peptide had been shown previously to readily accumulate intracellularly within cortical neurons and SH‐SY5Y cells at sublethal concentrations 17. This property was utilised to quantify colocalisation of phospho‐HSP20 and FAM‐Aβ_1–42_ using Pearson's correlation coefficient (PCC), a statistical tool used to correlate the spectral overlap between the red (phosph‐HSP20) and green (FAM‐Aβ_1–42_) emission channels 18. We observed a PCC value of 0.327 (±0.034 SEM) in cells treated with vehicle alone indicating little colocalisation (Fig. 6B). All PDE inhibitors tested induced significant increases in the colocalisation of FAM‐Aβ_1–42_ with phospho‐Hsp20 (Fig. 6B). Rolipram increased the PCC value to 0.458 (±0.04, *P* = 0.034), sildenafil to 0.426 (±0.03, *P* = 0.049), PF‐04447943 to 0.465 (±0.029, *P* = 0.017), and BAY 73‐6691 to 0.480 (±0.069, *P* = 0.022). These data suggest that increases in HSP20 phosphorylation through inhibition of PDE families 4, 5 and 9 serves to promote association of the chaperone with Aβ_1–42_ attenuating aggregation of the peptide and conferring protection against Aβ_1–42_ toxicity.

## Discussion

Cyclic nucleotides (cAMP and cGMP) are ubiquitous second messengers that are known to relay signals within the brain, influencing synaptic plasticity. In the context of AD, increases in cAMP and cGMP triggered by selective inhibition of PDE4 19, PDE5 20 and PDE9 21 have been responsible for improved synaptic function and memory in AD rodent models. Protective mechanisms promoted by increases in cAMP/cGMP include phosphorylation of the transcription factor CREB by PKA/PKG, which in turn promotes CREB binding to cAMP response elements on specific DNA sequences. This action activates transcription of genes involved in synaptic plasticity and neuronal protection, reversing Aβ_1–42_‐induced cognitive deficits (reviewed in 4).

Another protein that can be phosphorylated by PKA/PKG and confers protection in AD is the small heatshock protein HSP20. Phosphorylation of HSP20 is known to initiate a variety of cardio‐protective effects 8, 22 and promote protection against ischaemia–reperfusion injury in the brain via a mechanism involving modulation of Bcl‐2 and Bax expression 23. However, the chaperone's neuroprotective abilities in AD had not previously been linked with its phosphorylation. Recent work from our laboratory has demonstrated that phosphorylation of HSP20 by PKA increases its association with the aggregation domain of Aβ_1–42_, reducing oligomerisation 12 and neuronal toxicity of the peptide 13. We show here that PDE inhibitors, which target specifically PDEs 4, 5 and 9, induce the phosphorylation of HSP20 in a dose‐ and time‐dependent manner. It is already known that HSP20 exists in a complex with AKAP‐Lbc 24 and PDE4D 11, and that dissociation of PDE4 from this signalosome results in the phosphorylation of HSP20, which acts to attenuate hypertrophic signalling in a heart failure model 25. In contrast, the direct association of PDE5 and PDE9 with HSP20 has not been investigated, yet our data suggest that the activities of these PDEs also influence the probability of HSP20 phosphorylation. Indeed the phosphorylation of HSP20 is enhanced in coronary arteries following treatment with the PDE5 inhibitor sildenafil 26, but no such data exist for the inhibition of PDE9. In light of this, it was unexpected that the PDE9 inhibitors performed best in each assay within this study, producing the largest HSP20 phosphorylation, the greatest level of protection against Aβ‐induced cytotoxicity, and the most pronounced increase in colocalisation between phospho‐HSP20 and FAM‐Aβ_1–42_. Interestingly, PDE9 inhibitors have been investigated as modulators of the nitric oxide/guanylate cyclase/PKG signalling pathway as a therapeutic strategy 27, 28 and are currently under evaluation in clinical trials as cognitive enhancers for AD. We speculate that the molecular mechanisms underpinning the neuroprotective capacity of PDE9 inhibition may involve the enhanced HSP20 phosphorylation, although a recent study suggests that certain PDE9 inhibitors may also prevent Aβ_1–42_ oligomerisation by acting as metal ion chelators 29.

## Author contributions

GSB, AIP and RTC conceived the study; RTC and GSB designed experiments, analysed data and wrote the manuscript; RTC, JPD and EW performed experiments; GSB and EW made manuscript revisions.

## References

[1] Selkoe DJ and Hardy J (2016) The amyloid hypothesis of Alzheimer's disease at 25 years. EMBO Mol Med 8, 595–608. 27025652 10.15252/emmm.201606210 PMC4888851

[2] Lee D (2015) Global and local missions of cAMP signaling in neural plasticity, learning, and memory. Front Pharmacol 6, 161. 26300775 10.3389/fphar.2015.00161 PMC4523784

[3] Domek‐Lopacinska KU and Strosznajder JB (2010) Cyclic GMP and nitric oxide synthase in aging and Alzheimer's disease. Mol Neurobiol 41, 129–137. 20213343 10.1007/s12035-010-8104-x

[4] Garcia‐Osta A , Cuadrado‐Tejedor M , Garcia‐Barroso C , Oyarzabal J and Franco R (2012) Phosphodiesterases as therapeutic targets for Alzheimer's disease. ACS Chem Neurosci 3, 832–844. 23173065 10.1021/cn3000907 PMC3503343

[5] Saura CA and Valero J (2011) The role of CREB signaling in Alzheimer's disease and other cognitive disorders. Rev Neurosci 22, 153–169. 21476939 10.1515/RNS.2011.018

[6] Maurice DH , Ke H , Ahmad F , Wang Y , Chung J and Manganiello VC (2014) Advances in targeting cyclic nucleotide phosphodiesterases. Nat Rev Drug Discov 13, 290–314. 24687066 10.1038/nrd4228 PMC4155750

[7] Xu Y , Zhang HT and O'Donnell JM (2011) Phosphodiesterases in the central nervous system: implications in mood and cognitive disorders. Handb Exp Pharmacol 204, 447–485. 10.1007/978-3-642-17969-3_19 21695652

[8] Edwards HV , Cameron RT and Baillie GS (2011) The emerging role of AD as a multifunctional protective agent. Cell Signal 23, 1447–1454. 21616144 10.1016/j.cellsig.2011.05.009

[9] Wilhelmus MM , Otte‐Holler I , Wesseling P , de Waal RM , Boelens WC and Verbeek MM (2006) Specific association of small heat shock proteins with the pathological hallmarks of Alzheimer's disease brains. Neuropathol Appl Neurobiol 32, 119–130. 16599941 10.1111/j.1365-2990.2006.00689.x

[10] Wilhelmus MM , Boelens WC , Otte‐Holler I , Kamps B , de Waal RM and Verbeek MM (2006) Small heat shock proteins inhibit amyloid‐beta protein aggregation and cerebrovascular amyloid‐beta protein toxicity. Brain Res 1089, 67–78. 16635482 10.1016/j.brainres.2006.03.058

[11] Sin YY , Edwards HV , Li X , Day JP , Christian F , Dunlop AJ , Adams DR , Zaccolo M , Houslay MD and Baillie GS (2011) Disruption of the cyclic AMP phosphodiesterase‐4 (PDE4)‐HSP20 complex attenuates the beta‐agonist induced hypertrophic response in cardiac myocytes. J Mol Cell Cardiol 50, 872–883. 21334344 10.1016/j.yjmcc.2011.02.006

[12] Quinn SD , Dalgarno PA , Cameron RT , Hedley GJ , Hacker C , Lucocq JM , Baillie GS , Samuel ID and Penedo JC (2014) Real‐time probing of beta‐amyloid self‐assembly and inhibition using fluorescence self‐quenching between neighbouring dyes. Mol BioSyst 10, 34–44. 24170094 10.1039/c3mb70272c

[13] Cameron RT , Quinn SD , Cairns LS , MacLeod R , Samuel ID , Smith BO , Carlos Penedo J and Baillie GS (2014) The phosphorylation of Hsp20 enhances its association with amyloid‐beta to increase protection against neuronal cell death. Mol Cell Neurosci 61, 46–55. 24859569 10.1016/j.mcn.2014.05.002 PMC4148482

[14] Lambert MP , Barlow AK , Chromy BA , Edwards C , Freed R , Liosatos M , Morgan TE , Rozovsky I , Trommer B , Viola KL *et al* (1998) Diffusible, nonfibrillar ligands derived from Abeta1‐42 are potent central nervous system neurotoxins. Proc Natl Acad Sci USA 95, 6448–6453. 9600986 10.1073/pnas.95.11.6448 PMC27787

[15] Diemert S , Dolga AM , Tobaben S , Grohm J , Pfeifer S , Oexler E and Culmsee C (2012) Impedance measurement for real time detection of neuronal cell death. J Neurosci Methods 203, 69–77. 21963366 10.1016/j.jneumeth.2011.09.012

[16] Hsu YY , Liu CM , Tsai HH , Jong YJ , Chen IJ and Lo YC (2010) KMUP‐1 attenuates serum deprivation‐induced neurotoxicity in SH‐SY5Y cells: roles of PKG, PI3K/Akt and Bcl‐2/Bax pathways. Toxicology 268, 46–54. 19962417 10.1016/j.tox.2009.11.021

[17] Hu X , Crick SL , Bu G , Frieden C , Pappu RV and Lee JM (2009) Amyloid seeds formed by cellular uptake, concentration, and aggregation of the amyloid‐beta peptide. Proc Natl Acad Sci USA 106, 20324–20329. 19910533 10.1073/pnas.0911281106 PMC2787156

[18] Zinchuk V , Zinchuk O and Okada T (2007) Quantitative colocalization analysis of multicolor confocal immunofluorescence microscopy images: pushing pixels to explore biological phenomena. Acta Histochem Cytochem 40, 101–111. 17898874 10.1267/ahc.07002 PMC1993886

[19] Cheng YF , Wang C , Lin HB , Li YF , Huang Y , Xu JP and Zhang HT (2010) Inhibition of phosphodiesterase‐4 reverses memory deficits produced by Abeta25‐35 or Abeta1‐40 peptide in rats. Psychopharmacology (Berl) 212, 181–191. 20640406 10.1007/s00213-010-1943-3

[20] Puzzo D , Staniszewski A , Deng SX , Privitera L , Leznik E , Liu S , Zhang H , Feng Y , Palmeri A , Landry DW *et al* (2009) Phosphodiesterase 5 inhibition improves synaptic function, memory, and amyloid‐beta load in an Alzheimer's disease mouse model. J Neurosci 29, 8075–8086. 19553447 10.1523/JNEUROSCI.0864-09.2009 PMC6666028

[21] Kroker KS , Mathis C , Marti A , Cassel JC , Rosenbrock H and Dorner‐Ciossek C (2014) PDE9A inhibition rescues amyloid beta‐induced deficits in synaptic plasticity and cognition. Neurobiol Aging 35, 2072–2078. 24746365 10.1016/j.neurobiolaging.2014.03.023

[22] Fan GC and Kranias EG (2011) Small heat shock protein 20 (HspB6) in cardiac hypertrophy and failure. J Mol Cell Cardiol 51, 574–577. 20869365 10.1016/j.yjmcc.2010.09.013 PMC3033453

[23] Zeng L , Tan J , Lu W and Hu Z (2013) Blockade of Ser16‐Hsp20 phosphorylation attenuates neuroprotection dependent upon Bcl‐2 and Bax. Curr Neurovasc Res 10, 208–215. 23713735 10.2174/15672026113109990006

[24] Edwards HV , Scott JD and Baillie GS (2012) The A‐kinase‐anchoring protein AKAP‐Lbc facilitates cardioprotective PKA phosphorylation of Hsp20 on Ser(16). Biochem J 446, 437–443. 22731613 10.1042/BJ20120570 PMC3498943

[25] Martin TP , Hortigon‐Vinagre MP , Findlay JE , Elliott C , Currie S and Baillie GS (2014) Targeted disruption of the heat shock protein 20‐phosphodiesterase 4D (PDE4D) interaction protects against pathological cardiac remodelling in a mouse model of hypertrophy. FEBS Open Bio 4, 923–927. 10.1016/j.fob.2014.10.011 PMC4239479 25426411

[26] Tessier DJ , Komalavilas P , McLemore E , Thresher J and Brophy CM (2004) Sildenafil‐induced vasorelaxation is associated with increases in the phosphorylation of the heat shock‐related protein 20 (HSP20). J Surg Res 118, 21–25. 15093712 10.1016/j.jss.2004.01.001

[27] Zhihui Q (2013) Modulating nitric oxide signaling in the CNS for Alzheimer's disease therapy. Future Med Chem 5, 1451–1468. 23919554 10.4155/fmc.13.111

[28] Verhoest PR , Fonseca KR , Hou X , Proulx‐Lafrance C , Corman M , Helal CJ , Claffey MM , Tuttle JB , Coffman KJ , Liu S *et al* (2012) Design and discovery of 6‐[(3S,4S)‐4‐methyl‐1‐(pyrimidin‐2‐ylmethyl)pyrrolidin‐3‐yl]‐1‐(tetrahydro‐2H‐pyr an‐4‐yl)‐1,5‐dihydro‐4H‐pyrazolo[3,4‐d]pyrimidin‐4‐one (PF‐04447943), a selective brain penetrant PDE9A inhibitor for the treatment of cognitive disorders. J Med Chem 55, 9045–9054. 22780914 10.1021/jm3007799

[29] Su T , Zhang T , Xie S , Yan J , Wu Y , Li X , Huang L and Luo HB (2016) Discovery of novel PDE9 inhibitors capable of inhibiting Abeta aggregation as potential candidates for the treatment of Alzheimer's disease. Sci Rep 6, 21826. 26911795 10.1038/srep21826 PMC4766439

